# Editorial: Control of Regulatory T Cell Stability, Plasticity, and Function in Health and Disease

**DOI:** 10.3389/fimmu.2020.611591

**Published:** 2021-01-27

**Authors:** Silvia Piconese, Lucy S. K. Walker, Margarita Dominguez-Villar

**Affiliations:** ^1^Department of Internal Clinical Sciences, Anaesthesiology and Cardiovascular Sciences, Sapienza University of Rome, Rome, Italy; ^2^Laboratory Affiliated to Istituto Pasteur Italia - Fondazione Cenci Bolognetti, Rome, Italy; ^3^Institute of Immunity and Transplantation, University College London Division of Infection and Immunity, Royal Free Campus, London, United Kingdom; ^4^Faculty of Medicine, Department of Infectious Diseases, Imperial College London, London, United Kingdom

**Keywords:** regulatory T cell (T reg), plasticity, autoimmunity, cancer, metabolism

Regulatory T cells (CD4^+^CD25^high^CD127^−^FOXP3^+^, Tregs) play a fundamental role in maintaining immune homeostasis by modulating the immune response against self-antigens, allergens, pathogens, and tumors. While Tregs were originally thought to be a terminally differentiated population of T cells whose only function was to inhibit the activation and/or proliferation of other immune cells, studies over the past decade have established that Tregs are a more plastic and dynamic population than previously thought and they have a far broader role mediated by their interaction with several immune and non-immune cells.

This Research Topic contains contributions that address the molecular mechanisms that regulate Foxp3 expression and Treg function, plasticity and instability, and the influence of environmental factors on these mechanisms in health and disease.

Tregs are a fairly stable population under homeostatic conditions, with tight regulation of the two major axes that establish the Treg program, i.e., Foxp3 expression and a Treg-specific epigenetic signature that is acquired during Treg development in the thymus (1). Stable and long-term expression of Foxp3 in Tregs is essential for Treg function and is partly controlled by the demethylation of Treg-specific epigenetic signature genes, including Foxp3 (2). Herppich et al. examine the dynamics of the imprinting of Treg-specific epigenetic signature genes in thymic-derived Tregs and demonstrate that the establishment of the Treg cell-specific hypomethylation patterns is a continuous process throughout thymic Treg development.

Regarding the regulation of Foxp3 expression and function, Zhang and Zhou review the evidence on Treg instability and the intrinsic and extrinsic mechanisms that control Foxp3 expression, proposing an interesting hypothesis that Foxp3 instability might help thymic derived Tregs distinguish between self and non-self antigens. In addition, Deng et al. review the post-translational modifications that control Foxp3 protein expression and therefore, Treg function, and Colamatteo et al. review the mechanisms that control Foxp3 expression in healthy and autoimmune conditions.

Under inflammatory conditions, some Tregs can produce pro-inflammatory cytokines such as IFNγ and acquire an aberrant effector-like phenotype (plasticity) (3, 4) or even lose Foxp3 expression (instability) (5, 6). Such changes can be triggered in diverse settings including autoimmune, allergic, and infectious diseases (7). For example, patients with relapsing-remitting multiple sclerosis (MS), who display an increased frequency of IFNγ-producing Th1-like Tregs and a decrease in Treg suppressive function (3). Furthermore, a small percentage of Tregs in mouse models of MS have been shown to lose Foxp3 expression and become effector T cells, producing pro-inflammatory cytokines (IFNγ and IL-17) and contributing to disease severity (5, 6). In this regard, two reports in this book identify additional factors necessary to maintain Treg stability. Ronin et al. report that mice with a specific deletion of RelA in Tregs develop autoimmunity, which is attributable to the role of RelA in promoting Treg activation and stability, as RelA knock out Tregs lose Foxp3 expression and produce pro-inflammatory cytokines.

Di Giovangiulio et al. examine the involvement of the Tbet-IFNγ axis in colitis development. Tregs isolated from the lamina propria of active IBD patients display a Th1-like phenotype. Using a conditional Treg-specific Tbet KO, they observe that Tbet expression in Tregs is required for the development of colitis, and mice with Tbet KO Tregs develop milder colitis.

While these Treg plasticity and instability events are controlled by intrinsic molecular signaling pathways such as the PI3K/AKT pathway (8, 9), the activation of such pathways is modulated by the Treg environment, including cytokines, metabolic intermediates and dietary factors. As examples, Bin Dhuban et al. demonstrate that IL-27 and IL-6 signaling *via* gp130 impair the suppressive capacity of Tregs and render these Tregs unstable by downregulating Helios. Urbano et al. show that TNFα signaling through TNFR2 regulates the kinase architecture of activated Tregs and controls the expression of IL-17. Zhou et al. study the involvement of the HMGB1/PTEN/β-catenin pathway in myeloid cells in the development of Tregs during acute lung injury.

In regards to the influence of metabolism on Treg stability, Kempkes et al. review the metabolic profiles associated with the regulation of Treg functionality, and Shi and Chi provide a summary on the extrinsic and intrinsic metabolic factors and programs that regulate Treg lineage stability and plasticity, both in lymphoid and non-lymphoid tissues. Moreover, Arroyo Hornero et al. discuss the mechanisms underlying the effects of certain dietary components, including NaCl and fatty acids, on modulating Treg stability, plasticity, and function.

The tumor microenvironment is responsible for the specific phenotypes and functionality of infiltrating immune cells (10, 11). Toor et al. phenotypically characterize Tregs infiltrating tumor tissue in colorectal cancer patients and compare them to normal colon tissues and peripheral blood. They find an enrichment of highly suppressive Tregs (Foxp3^+^Helios^+^) in the tumor microenvironment, which upregulate a number of inhibitory receptors including CTLA-4, TIM-3, PD-1, and LAG-3. These receptors have been shown to modulate Treg function, migration, and proliferation (12).

Tregs can interact with non-immune cells and populate non-lymphoid tissues, where they perform non-immunological functions, mostly related to tissue repair and organ homeostasis (13). Korn and Muschaweckh and Sambucci et al. discuss the mechanisms that maintain Treg stability and function in non-lymphoid tissues, utilizing the central nervous system in the context of autoimmunity as an example. Albany et al. review the involvement of Tregs in cardiovascular disease and atherosclerosis and Brown et al. discuss the mechanisms that drive Treg adaptation to the environment and host tissues. In a perspective article, Martini et al. propose the intriguing hypothesis that Tregs have evolved under the pressure of mammalian pregnancy and lactation and tolerization to mammalian gut microflora. However, the Treg benefit turns into unwanted deleterious effects at advanced ages, when autoimmune and cardiovascular diseases and cancer can develop.

Tregs are a heterogeneous population and the biology underlying specific subpopulations is important to understand different roles of Tregs in various diseases. For example, Ding et al. give an update on follicular regulatory T cell biology with a particular focus on autoimmunity. Motwani et al. perform a deep characterization of cord blood *versus* adult blood Tregs and demonstrate that adult blood Tregs are a more heterogeneous population with less effector-like cells, and point to the use of expanded cord blood Tregs as a potential optimal adoptive cell therapy option for the treatment of autoimmune and inflammatory diseases.

Altogether, these works provide a comprehensive update on the immunological mechanisms underlying the control of Treg functionality, plasticity and instability, and the involvement of environmental factors in their modulation. This novel information on control of Treg stability, plasticity and function could help to guide the development of novel therapies to treat human disease.

## Author Contributions

All authors listed have made a substantial and direct intellectual contribution to the work, and all approved it for publication.

## Conflict of Interest

The authors declare that the research was conducted in the absence of any commercial or financial relationships that could be construed as a potential conflict of interest.
